# DNA methylation inhibitor decitabine ameliorates spondyloarthritis by suppressing Th17 responses and epigenetically upregulating VIPR1 in SKG mice

**DOI:** 10.1186/s13075-026-03848-0

**Published:** 2026-06-18

**Authors:** Hoshiko Furusawa, Goh Murayama, Masaki Nojima, Taiga Kuga, Yukitomo Hagiwara, Yoshiyuki Abe, Makio Kusaoi, Kurisu Tada, Ken Yamaji, Naoto Tamura

**Affiliations:** Department of Internal Medicine and Rheumatology, Juntendo University Faculty of Medicine, 2-1-1 Hongo, Bunkyo-ku, Tokyo, 113-8421 Japan

**Keywords:** Spondyloarthritis, DNA methylation, Decitabine, Th17, Vipr1, SKG mouse model

## Abstract

**Background:**

Spondyloarthritis (SpA) is a chronic inflammatory disorder characterized by enthesitis of axial and peripheral joints. Although genetic factors, bacterial infection, dysbiosis, and mechanical stress contribute to its development, the role of epigenetic regulation remains poorly understood. DNA methylation has been implicated in immune dysregulation, and the DNA methyltransferase inhibitor, decitabine (DAC), has demonstrated therapeutic effects in autoimmune disease models. This study aimed to evaluate the effect of DAC in the SpA-resembling SKG/Jcl (SKG) mouse model and elucidate the immunological mechanisms.

**Methods:**

SpA-like arthritis was induced in SKG mice via intraperitoneal injection of curdlan (1,3-β-glucan aggregates). DAC (1 mg/kg) was administered weekly. Arthritis severity and histopathological changes were evaluated in the limb joints and tail, and histopathological enthesitis scores were assessed in the limb joints. Splenic T-cell profiles were analyzed by flow cytometry. CD4⁺ T cells were stimulated in vitro with DAC to assess cytokine production. To investigate epigenetic regulation, reduced representation bisulfite sequencing (RRBS) and RNA-seq were performed on Achilles tendon tissue containing enthesis. Local expression of IL-17 and vasoactive intestinal peptide receptor 1 (VIPR1) in peripheral entheses was assessed by immunohistochemistry.

**Results:**

DAC treatment significantly reduced arthritis severity and improved histopathological findings, including synovial hyperplasia, inflammatory cell infiltration, bone erosion, and cartilage loss. Peripheral enthesitis at the Achilles tendon insertion sites was also reduced. Flow cytometric analysis demonstrated that the DAC-treated group reduced IL-17 A expression in splenic CD4⁺ T cells during the early phase (weeks 4–8) and decreased PD-1 expression in regulatory T cells (Tregs) at week 12. In vitro, DAC suppressed IL-17 A secretion and enhanced TGF-β production in stimulated splenic CD4⁺ T cells. Integrated RRBS and RNA-seq analyses identified *Vipr1* as a DAC-responsive gene, showing significant hypomethylation (Δmethylation ≥ − 25%) and concordant transcriptional upregulation (log₂FC > 1) in the entheses of DAC-treated mice. Immunohistochemical analysis of the ankle entheses revealed decreased IL-17 A expression and increased VIPR1 expression.

**Conclusions:**

DAC ameliorated SpA in SKG mice, possibly through suppression of Th17 responses and epigenetic upregulation of *Vipr1*, and may be associated with improved Treg-associated regulation. These findings highlight an important role of DNA methylation in SpA pathogenesis.

**Supplementary Information:**

The online version contains supplementary material available at 10.1186/s13075-026-03848-0.

## Background

Spondyloarthritis (SpA) is a group of rheumatic diseases characterized by chronic enthesitis and arthritis of axial and peripheral joints, leading to substantial impairment in the quality of life of patients [1]. Its pathogenesis is complex, involving interactions between genetic—especially *HLA-B27* in axial SpA—and environmental (including bacterial infection, dysbiosis, and mechanical stress) factors; however, the underlying mechanisms remain incompletely understood [2, 3]. Although axial SpA shows a strong genetic association with *HLA-B27*, its occurrence in *HLA-B27*-negative individuals suggests the involvement of non-genetic mechanisms, including epigenetic regulation [4, 5]. SpA is mainly driven by the interleukin (IL)-17/IL-23 pathway, and recent advances in molecular targeted therapies, including tumor necrosis factor (TNF), IL-17, and Janus kinase inhibitors, have markedly improved clinical outcomes in patients with SpA [6–8]. However, some patients exhibit inadequate response, diminished efficacy over long-term treatment, or drug-related adverse effects. Therefore, elucidating the underlying mechanisms of SpA pathogenesis and developing novel therapeutic strategies based on disease-driving pathways remains essential.

In recent years, increasing attention has focused on the role of epigenetic regulation—particularly DNA methylation—in the onset and progression of rheumatic diseases. DNA methylation is a key epigenetic mechanism that regulates gene expression by adding methyl groups to cytosine residues in DNA, thereby suppressing gene transcription [9]. Aberrant DNA methylation patterns have been reported in immune cells, such as T and B cells, from patients with rheumatic diseases, including systemic lupus erythematosus, type 1 diabetes mellitus, rheumatoid arthritis, Graves’ disease, and Hashimoto’s thyroiditis, suggesting their involvement in disease development and progression [10, 11]. Decitabine (DAC), a DNA methylation inhibitor that blocks DNA methyltransferase activity, is clinically used in some countries to treat myelodysplastic syndrome and alleviates disease severity in experimental models of multiple sclerosis, type 1 diabetes, and collagen-induced arthritis [12, 13]. Mechanistically, DAC inhibits abnormal DNA methylation and promotes FOXP3 expression, thereby inducing and enhancing regulatory T cell (Treg) function while suppressing Th1/Th17 differentiation [14–16].

The SKG mouse strain, which harbors a point mutation in ZAP-70—a tyrosine kinase associated with the T-cell receptor signaling complex—develops arthritis, vertebral disease, skin disease, and enteritis closely resembling human SpA following intraperitoneal injection of β-glucan-containing reagents. Mechanistically, β-glucans bind to the dectin-1 receptor on myeloid cells, activate SYK and CARD9 signaling, and subsequently induce IL-23 production via NF-κB activation. Consequently, self-reactive CD4⁺ T cells—whose autoreactivity is enhanced by the ZAP-70 mutation—differentiate into Th17 cells. Concurrently, defective Tregs fail to suppress Th17-mediated inflammation, leading to chronic IL-17-, TNF-, and GM-CSF-driven tissue damage in peripheral organs [17, 18].

Despite accumulating evidence linking epigenetic dysregulation to autoimmune diseases, the role of DNA methylation and the therapeutic potential of DNA methylation inhibitors in SpA remain largely unexplored. Therefore, this study aimed to comprehensively evaluate the therapeutic effects of DAC in a murine model of SpA. In this study, we demonstrated that DAC inhibits Th17 responses and partially restores Treg activity in a SpA pathogenesis. These effects are associated with the modulation of DNA methylation. Specifically, methylome–transcriptome analysis identified *Vipr1*, a known negative regulator of stromal inflammation and Th17 responses, as a key target epigenetically upregulated by DAC. Our findings provide mechanistic insight into targeting the epigenetic landscape as a promising therapeutic strategy for SpA.

## Methods

### Aim, design, and setting

This study aimed to determine the therapeutic effects of DAC and to elucidate its immunological and epigenetic mechanisms in a murine model of spondyloarthritis. We conducted a controlled longitudinal preclinical experimental study in curdlan-induced SKG mice administered DAC or vehicle control. Disease progression and treatment responses were evaluated using clinical arthritis scoring, histopathological assessment of joints and entheses, flow cytometric immune profiling, and integrated methylome–transcriptome analyses of Achilles tendon tissue containing the enthesis. All animal experiments were performed under specific pathogen–free conditions at the laboratory animal facility of Juntendo University School of Medicine in accordance with institutional guidelines for animal care and use.

### Mice

Female BALB/c SKG/Jcl mice (6–10 weeks old) were purchased from CLEA Japan, Inc. (Tokyo, Japan) and maintained under specific pathogen-free conditions with ad libitum access to food and water. All animal experiments were performed in accordance with the guidelines of the National Institutes of Health and approved by the Animal Experimentation Committee of Juntendo University School of Medicine. All procedures complied with institutional regulations for animal care and use.

### Experimental schedule

Female SKG mice (7–10 weeks old at the start of experiments) were intraperitoneally injected with 3 mg of curdlan (Wako, Japan) suspended in 1 mL phosphate-buffered saline (PBS) or PBS alone at weeks 0 and 2. All mice were monitored for up to 12 weeks. DAC was dissolved in dimethyl sulfoxide and diluted with PBS before injection. Mice received intraperitoneal injections of DAC (1 mg/kg; Apollo Scientific, Manchester, UK) or vehicle alone once weekly. The number of mice used varied depending on the experimental purpose, and separate cohorts were used for different analyses. The number of animals (n) for each experiment is indicated in the corresponding figure legends.

### Peripheral arthritis scoring

Clinical arthritis was evaluated every 2 weeks based on a previously described scoring system [19] ( 0 = no swelling or redness; 0.1 = swelling or redness of one digit; 0.5 = mild swelling and/or redness of the wrist or ankle joint; 1 = severe swelling of large joints).

### Histopathological scoring

Mice were euthanized 12 weeks after curdlan injection. Tissues from the hand and foot joints, as well as the spine, including the tail vertebrae, were fixed in 10% formalin, decalcified in ethylenediaminetetraacetic acid, and embedded in paraffin. Sections were deparaffinized in xylene, dehydrated through graded alcohols, and stained with hematoxylin–eosin (HE) and toluidine blue (TB). Bone erosion, cartilage loss (reduced staining intensity), synovial hyperplasia (multilayering), and inflammatory cell infiltration were graded on a scale of 0‒4 [20, 21]. Based on previously published criteria, peripheral enthesitis was graded on a 1–4 scale: 1, mild inflammation at the tendon insertion site; 2, mild-to-moderate inflammatory infiltrate at the insertion site and along the tendon; 3, severe inflammation with bone involvement; and 4, severe inflammation with obliteration of the tendon–bone interface [22].

### Flow cytometric analysis of splenocytes

For flow cytometric analysis, spleens were collected from mice sacrificed at 4, 8, and 12 weeks. Splenocytes were isolated by homogenization, and red blood cells were lysed using ammonium chloride potassium buffer. Cells were stained with the following monoclonal antibodies: anti-CD3ε–BV605, anti-CD4–FITC, anti-CD8–BV421 or PE, anti-CD45–BV510, anti-CD69–Alexa700, anti-CD44–APC, anti-CD62L–PE, anti-PD-1–PerCP, anti-CD185 (CXCR5)–BV785, anti-ICOS–BV785, anti-CD25–APC, and anti-CD19/F4/80 (all from BioLegend, San Diego, CA, USA). After surface staining, intracellular staining was performed using anti-IL-17 A–PE/Cy7, anti-TNFα–APC, anti-IFNγ–Alexa700 (BioLegend), and anti-FOXP3–BV421 (eBioscience, San Diego, CA, USA). Data were acquired using a BD LSRFortessa (BD Biosciences, San Jose, CA, USA). Cell subsets and mean fluorescence intensity were analyzed using FlowJo software (Tree Star Inc, Ashland, OR, USA). Detailed gating strategy for intracellular cytokine and Treg staining is provided in Supplementary File 1.

### In vitro co-culture of splenic CD4 ⁺ with DAC

CD4⁺ T cells were isolated from mouse spleens by negative selection using the CD4⁺ T Cell Isolation Kit (Miltenyi Biotec, Bergisch Gladbach, Germany) based on the protocol of the manufacturer. Isolated CD4⁺ T cells (5 × 10⁵ cells/well) were stimulated for 48 h with Dynabeads™ Mouse T-Activator CD3/CD28 (Thermo Fisher Scientific, Waltham, MA, USA) and recombinant mouse IL-2 (Roche Holding AG, Basel, Switzerland) in TEX Max medium (Miltenyi Biotec). Subsequently, cells were co-cultured with or without DAC (5 µM) for an additional 48 h. After 96 h of total culture, supernatants were collected, and TGF-β levels were measured using ELISA kits (Proteintech, Rosemont, IL, USA) based on the instructions of the manufacturer. Treg populations were analyzed by flow cytometry.

In a separate set of experiments conducted under the same culture conditions, cells were further stimulated with PMA/ionomycin (BioLegend) for 48 h, and IL-17 A levels in the supernatants were assessed by ELISA (Proteintech, Rosemont, IL, USA).

### Epigenomic and transcriptomic analysis

#### DNA methylation analysis (reduced representation bisulfite sequencing [RRBS])

Genomic DNA was extracted from frozen mouse Achilles tendon tissue, including the enthesis, and subjected to RRBS. Libraries were prepared using the Zymo-Seq RRBS Library Kit (Zymo Research, Irvine, CA, USA) and sequenced on an Illumina NovaSeq X Plus (Illumina, San Diego, CA, USA) platform (150 bp paired-end), generating approximately 60 million reads per sample. Raw reads were assessed with FastQC (Babraham Bioinformatics, Cambridge, UK) and trimmed using Trim Galore, followed by alignment to the mouse reference genome (mm10) with Bismark (Babraham). Methylation calling and coverage normalization were performed using methylKit (Bioconductor, Boston, MA, USA). Differentially methylated cytosines (DMCs) and regions were identified using logistic regression or Fisher’s exact test (*p* < 0.05 and ≥25% methylation difference). 

#### RNA-seq analysis

Total RNA was isolated from the same tendon tissue and used for poly(A)-selected, strand-specific RNA-seq library preparation with the NEBNext Ultra II Directional RNA Library Prep Kit (New England Biolabs, Ipswich, MA, USA). Libraries were sequenced on an Illumina NovaSeq X Plus platform (150 bp paired-end), yielding approximately 40 million reads (~ 6 Gbp) per sample. After quality control with FastQC and trimming with Trimmomatic (Usadel Lab, Aachen, Germany), reads were aligned to the mm10 genome using HISAT2 (Johns Hopkins University, Baltimore, MD, USA) and quantified at the gene level using featureCounts (Walter and Eliza Hall Institute, Melbourne, Australia). Expression values were normalized to transcripts per million, and DESeq2-normalized counts were used for downstream analyses. Differentially expressed genes were identified using DESeq2 (Bioconductor) (|log_2_ fold change| > 1, *p* < 0.05, Benjamini–Hochberg correction). Integrated analysis based on gene information and correlation was conducted in R (Version 4.4.1) using the mixOmics package (University of Queensland, Brisbane, Australia, Version 6.28.0). Visualization, including the generation of Heatmaps and X-Y plots for integrated RNA-seq and DNA-seq results, was conducted using ggplot2 (RStudio, Boston, MA, USA, Version 3.5.1), ComplexHeatmap (Bioconductor, Version 2.20.0), EnrichedHeatmap (Bioconductor, Version 1.34.0), and igraph (Bioconductor, Version 2.1.1). Unless otherwise specified, all analyses were performed in R (v4.x) using default parameters. Full software versions are listed in Additional File X. RRBS and RNA-seq library preparation, sequencing, and initial bioinformatic processing were outsourced to a commercial service provider in Tokyo, Japan. RRBS and RNA sequencing were performed using Achilles tendon enthesis samples obtained from individual mice without pooling.

### Immunohistochemistry of mouse ankle entheses

Mouse ankle joints were harvested at the indicated time points and fixed in 20% neutral buffered formalin at 20–25 °C. The specimens were decalcified using EDT-X (FALMA Co., Ltd., Tokyo, Japan) for up to 7 days, followed by routine paraffin embedding. Serial Sect.  (3 μm thick) were prepared from paraffin blocks, deparaffinized in xylene, and rehydrated through graded ethanol.

Antigen retrieval was performed by heat-induced epitope retrieval using either citrate (pH 6.0) or Tris-EDTA (pH 9.0) buffer, followed by cooling to room temperature. Endogenous peroxidase activity was quenched with 0.3% hydrogen peroxide in methanol for 30 min, and nonspecific binding was blocked with 2.5% horse serum for 20 min.

Sections were incubated overnight at 4 °C with the following primary antibodies: anti-IL-17 A (Abcam, Cambridge, UK; 1:100) and anti-VIPR1 (Proteintech Group, Rosemont, IL, USA; 1:200) rabbit polyclonal antibodies. After washing, the sections were incubated with ImmPRESS^®^ HRP Anti-Rabbit IgG (Vector Laboratories, Burlingame, CA, USA; MP-7401) for 8 min at room temperature. Immunoreactivity was visualized using 3,3′-diaminobenzidine, followed by hematoxylin counterstaining.

Stained sections were dehydrated and mounted, and representative images of the ankle enthesis were acquired using an HS All-in-One fluorescence microscope (BZ-9000; KEYENCE, Osaka, Japan). No quantitative analysis was performed.

### Statistical analysis

All data were analyzed using GraphPad Prism (version 10.3.0) (GraphPad Software, San Diego, CA, USA). Statistical analyses were performed using Welch’s t-test for comparisons between two independent groups and Welch’s one-way analysis of variance followed by Dunnett’s T3 post hoc test for comparisons between three groups. For paired samples, where cells from the same mouse were categorized into two conditions (with or without compound treatment), paired t-tests were employed. A *P* value < 0.05 was considered statistically significant.

## Results

### DAC administration ameliorated the severity of arthritis

**Fig. 1:**
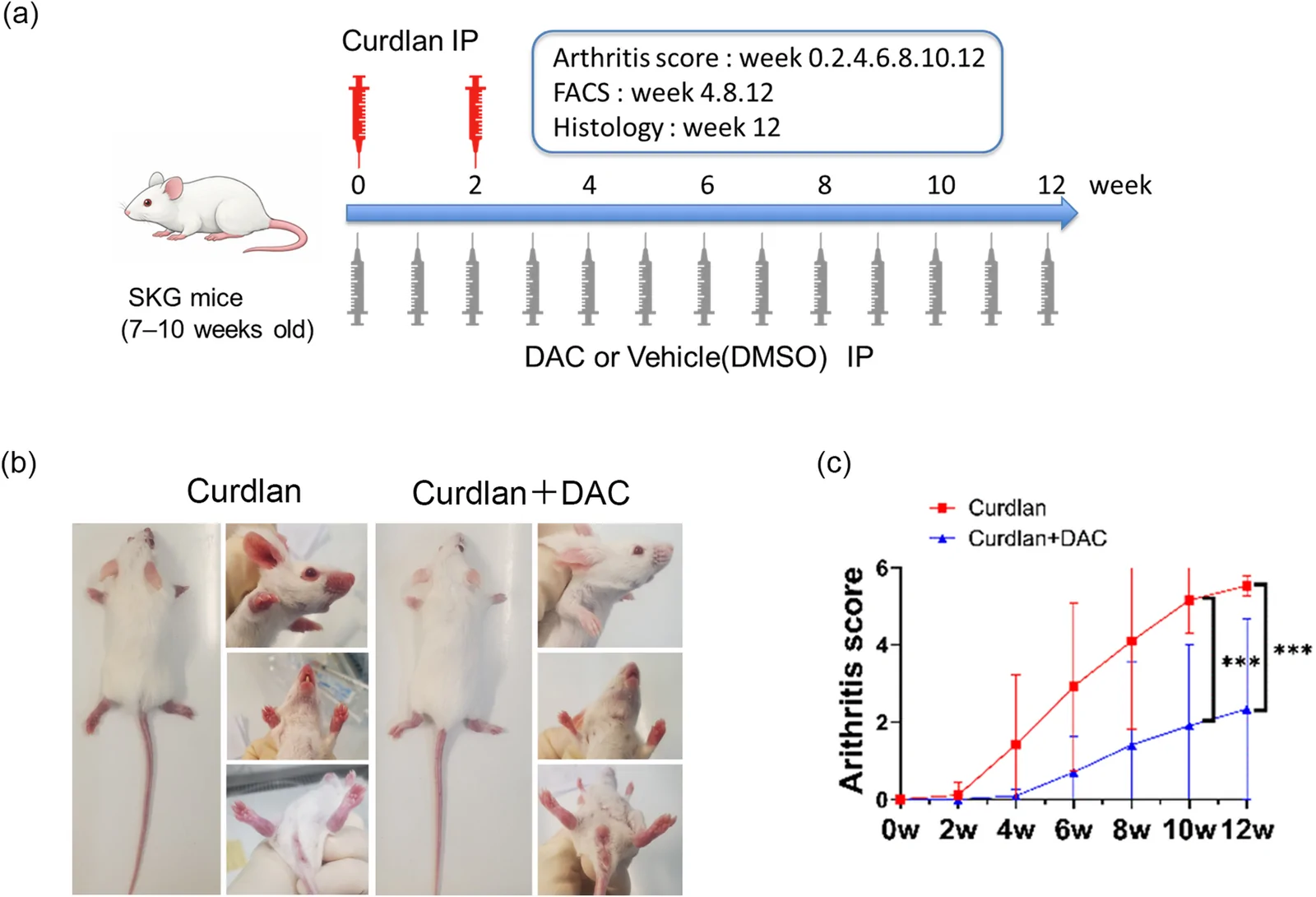
DAC administration ameliorated the severity of arthritis. **a** Experimental design. Curdlan-induced SKG mice were treated with DAC (1 mg/kg, i.p.) or vehicle once weekly and monitored for up to 12 weeks. **b** Representative images of wrist and ankle joints from vehicle-controled and DAC-treated SKG mice at week 12 after curdlan injection. **c** Arthritis scores over time in vehicle-treated and DAC-treated mice. Data represent mean ± SD (*n* = 10 per group). Groups were compared using Welch’s t-test. ***p < 0.0001. Abbreviations: DAC, decitabine; SD, standard deviation

SKG mice injected with curdlan developed progressive inflammatory arthritis affecting the joints of the limbs, digits, and tail. Inflammation initially involved the small joints of the ankles, wrists, or digits and subsequently extended to the tail, accompanied by redness and swelling. Periocular erythema and hair loss around the eyes and nose were also observed. Figure 1a shows representative images of arthritis at week 12 in vehicle-control and DAC-treated mice. Compared with vehicle-control mice, those receiving DAC exhibited markedly reduced joint swelling, periocular erythema, and hair loss. DAC administration significantly suppressed arthritis progression, resulting in lower arthritis scores at weeks 10 and 12 compared with vehicle-control mice (*n* = 10 per group) (Fig. 1b).

### DAC administration improved limb joints, tail, and peripheral enthesitis

**Fig. 2:**
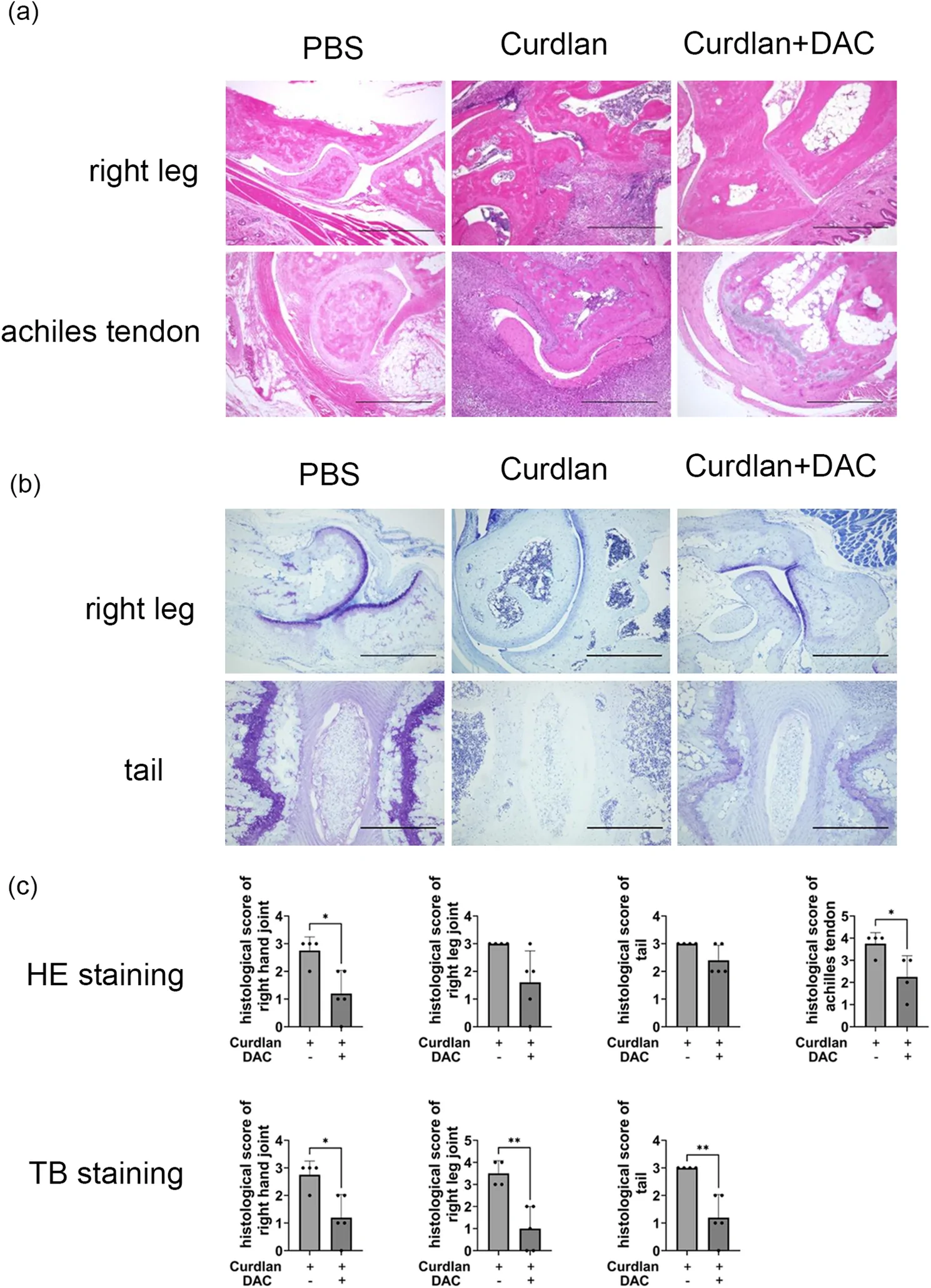
DAC administration improved limb joints, tail, and peripheral enthesitis. **a** HE staining of wrist and ankle joints. **b** TB staining of wrist joints, tarsal bones, tail, and Achilles tendon enthesis. **c** Histopathological scores based on HE and TB staining. Data represent mean ± SD (*n* = 5 per group). Groups were compared using Welch’s t-test. *p < 0.05; **p < 0.01. Scale bar = 500 μm. Abbreviations: DAC, decitabine; HE, hematoxylin–eosin; TB, toluidine blue; ANOVA, analysis of variance

Histopathological analysis was performed 12 weeks after curdlan injection. Figure 2a illustrates representative HE-stained sections, whereas Fig. 2b displays TB staining for each group. In the vehicle-control group, HE staining revealed marked synovial cell proliferation, inflammatory infiltration, and bone destruction around the wrist and tarsal joints. These pathological changes were notably suppressed in the DAC-treated group. In addition, inflammatory cell infiltration at the Achilles tendon enthesis was evident in vehicle-control mice and was markedly reduced in the DAC-treated group (Fig. 2a). Similarly, TB staining demonstrated cartilage matrix depletion and destruction in vehicle-control mice, whereas these pathological changes were less pronounced in the DAC-treated group. Figure 2c presents the histopathological scores for each group. HE-based scores were significantly lower in the wrist joints of DAC-treated mice and showed a trend toward lower scores in the ankle joints. TB-based scores were significantly lower in the limb joints and tail in the DAC-treated group. Furthermore, enthesitis scores at the Achilles tendon insertion site were also significantly lower in the DAC-treated group. (*n* = 5 per group)

### DAC suppresses splenic T-cell activation and Th17 responses in curdlan-induced SKG mice

**Fig. 3:**
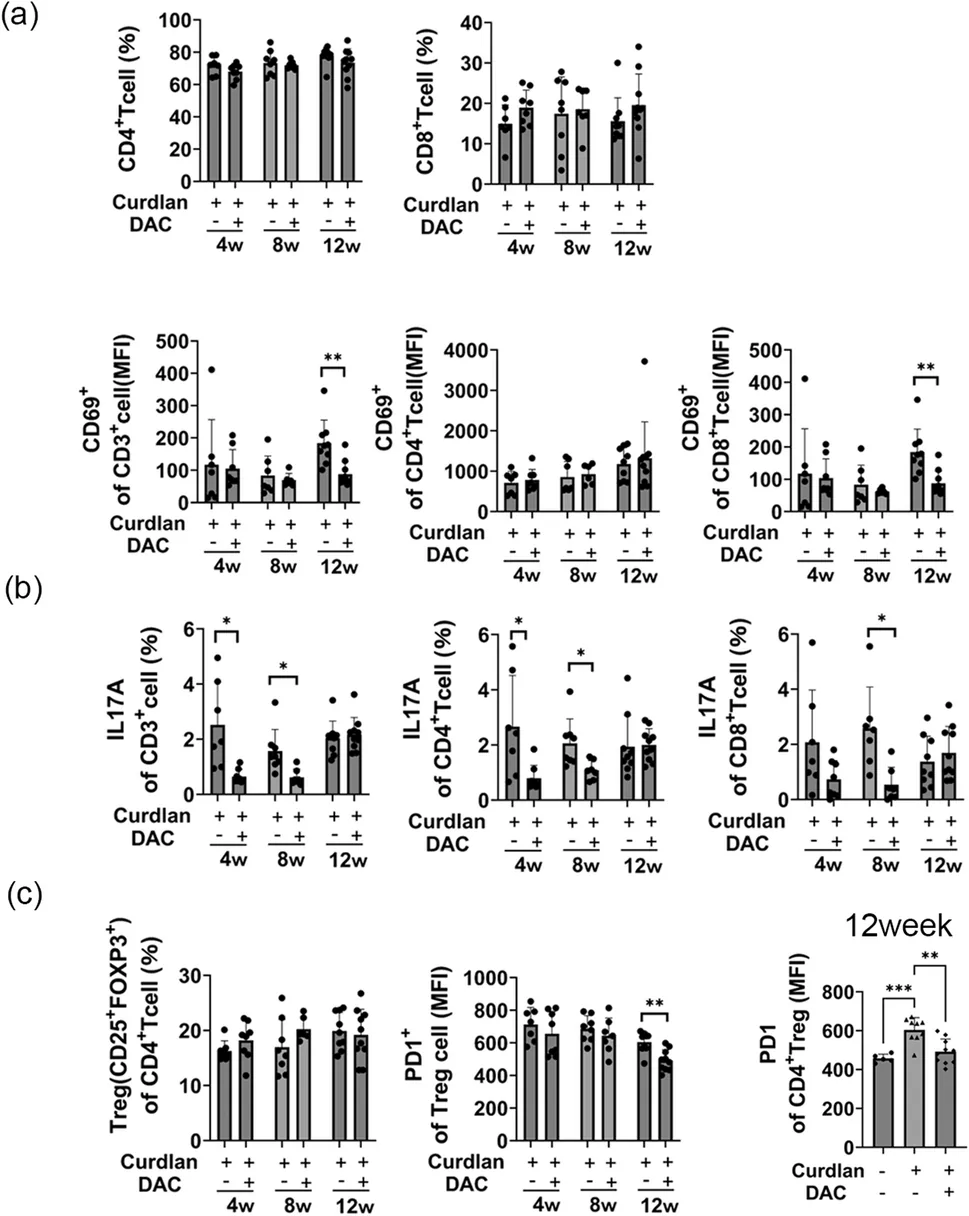
DAC suppresses splenic T-cell activation and Th17 responses in curdlan-induced SKG mice. Flow cytometric analysis of splenic T cells. **a** Frequencies of CD4⁺ and CD8⁺ T cells (expressed as a percentage of CD3⁺ T cells) and the MFI of CD69 expression on CD3⁺, CD4⁺ and CD8⁺ T cells are presented. **b** Intracellular cytokine staining showing the frequency of IL-17 A-positive cells among CD3⁺, CD4⁺, and CD8⁺ T cells. **c** Frequencies of regulatory T cells (CD4⁺CD25⁺Foxp3⁺) among CD4+ T cells and the MFI of PD-1 expression on these cells. (*n* = 8; weeks 4 and 8), (*n* = 4 for curdlan⁻/DAC⁻; *n* = 10 for curdlan⁺/DAC⁻ and curdlan⁺/DAC⁺ weeks 12). Data represent mean ± SD. Groups were compared using Welch’s t-test or Welch’s ANOVA(c-12week) as appropriate. *p < 0.05; **p < 0.01; ***p < 0.001. Abbreviations: DAC, decitabine; IL-17, interleukin-17; CD, cluster of differentiation; MFI, mean fluorescence intensity

To evaluate longitudinal changes in T-cell cytokine production, splenic cells collected at weeks 4, 8, and 12 after curdlan injection were analyzed via flow cytometry. At week 12, CD69 expression (mean fluorescence intensity, MFI) on CD3⁺ and CD8⁺ T cells was significantly reduced in the DAC-treated group (Fig. 3a). Intracellular staining revealed that IL-17 A production by CD3⁺ and CD4⁺ T cells was significantly suppressed in DAC-treated mice at weeks 4 and 8 (Fig. 3b). In addition to IL-17, we evaluated the effects of DAC on other cytokines. Interferon gamma (IFN-γ) production was significantly increased at week 4 following DAC treatment, whereas no consistent changes were observed in TNF-α levels (Supplementary Fig. 2).

Although the overall frequency of Tregs did not differ between groups, PD-1 expression (MFI) on Tregs tended to be lower in DAC-treated mice and was significantly reduced at week 12. Specifically, PD-1 expression (MFI) on Tregs was significantly elevated in the vehicle-control group compared with PBS-treated controls, whereas this increase was markedly attenuated in the DAC-treated group at week 12 (Fig. 3c).

### DAC modulates IL-17 A and Treg responses in CD4⁺ T cells from arthritic SKG mice in vitro

**Fig. 4:**
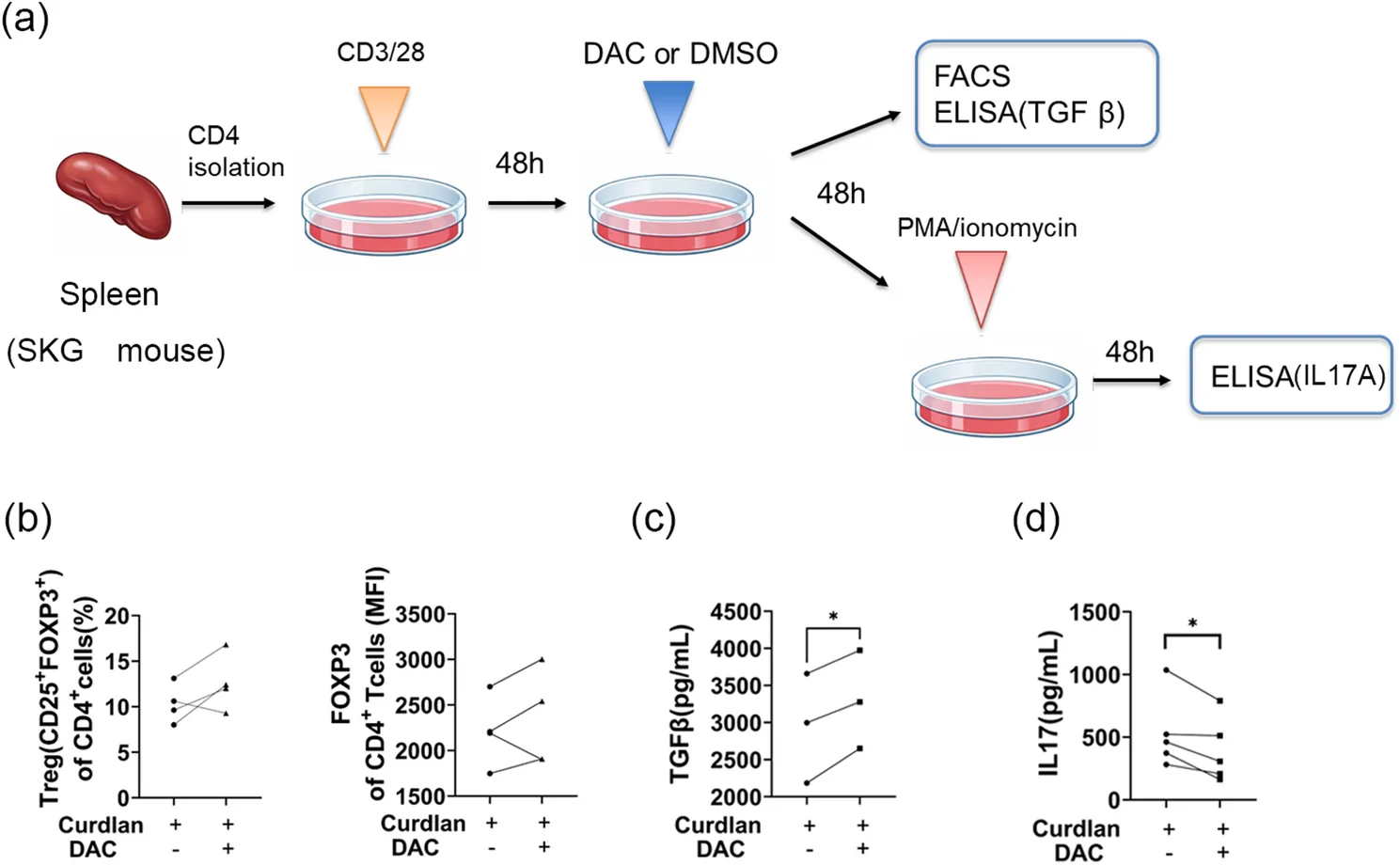
DAC modulates IL-17 and Treg responses in CD4⁺ T cells from arthritic SKG mice in vitro. **a** Experimental scheme of in vitro CD4⁺ T-cell stimulation and DAC treatment. CD4⁺ T cells isolated from the spleens of SKG mice 8 weeks after curdlan injection were stimulated for 48 h with CD3/CD28 in the presence of recombinant IL-2. Cells were further co-cultured with or without DAC (5 µM) for an additional 48 h. **b** Flowcytometric analysis of Treg frequency (CD4⁺CD25⁺Foxp3⁺) among CD4 T cells and Foxp3 expression (MFI) in CD4⁺ T-cell cultures. **c** TGF-β levels in culture supernatants. **d** IL-17 A production following PMA/ionomycin restimulation. Data represent mean ± SD. Paired comparisons were performed using paired t-tests. *p < 0.05. Abbreviations: DAC, decitabine; Tregs, regulatory T cells; PD-1, programmed cell death protein 1; TGF-β, transforming growth factor-beta; IL-17, interleukin-17; PMA, phorbol 12-myristate 13-acetate; MFI, mean fluorescence intensity

To confirm the effect of DAC, CD4⁺ T cells were isolated from splenocytes of mice 8 weeks after curdlan injection and cultured with DAC for 48 h. DAC treatment modestly increased the proportion of Tregs (CD4⁺CD25⁺FOXP3⁺) and FOXP3 (MFI),, although these changes did not reach statistical significance (Fig. 4a). Supernatant TGF-β levels were significantly increased in DAC-treated cultures compared with paired controls (Fig. 4b). Following additional stimulation with PMA/ionomycin, IL-17 A levels in the culture supernatants were significantly reduced in the presence of DAC, as determined by ELISA (Fig. 4c).

### DAC induces coordinated epigenomic and transcriptomic changes in the arthritic Achilles enthesis

**Fig. 5:**
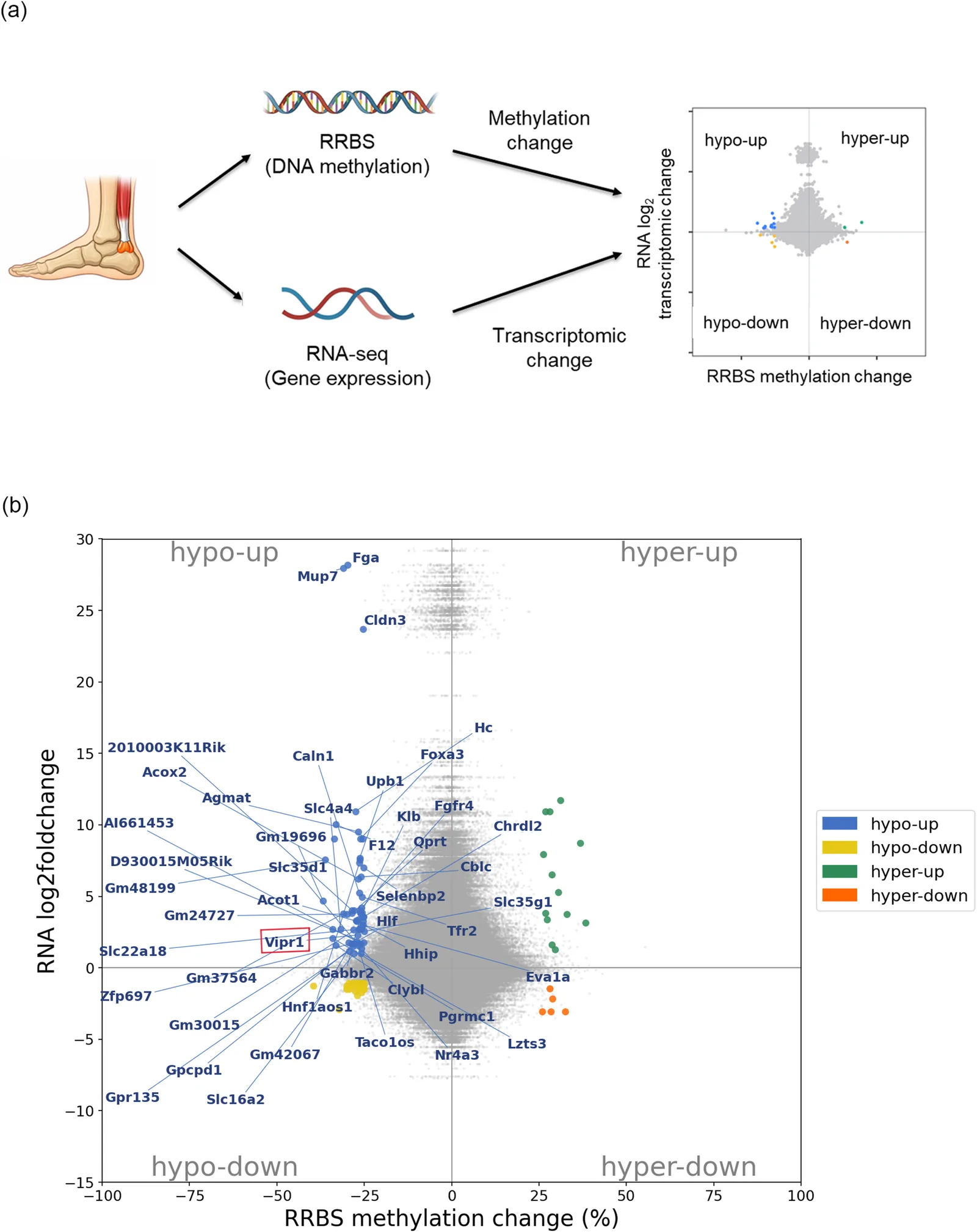
DAC induces coordinated epigenomic and transcriptomic changes in the arthritic Achilles enthesis. **a** Overview of the integrated RRBS and RNA-seq analysis workflow. Achilles tendon tissues from curdlan-induced SKG mice treated with vehicle or DAC were subjected to RRBS for DNA methylation profiling and RNA-seq for transcriptomic analysis, followed by integrated analysis to identify genes showing coordinated methylation and expression changes. **b** Volcano plot. Integrated X–Y plot showing the relationship between DNA methylation changes (x-axis; mean Δmethylation at differentially methylated cytosines [DMCs], ≥ 25% difference, *p* < 0.05) and gene expression changes (y-axis; log₂ fold change, |log₂FC| > 1, *p* < 0.05). Genes reaching these significance thresholds are color-coded and categorized into four groups based on the direction of methylation and transcriptional changes. DAC treatment predominantly enriched genes exhibiting concurrent hypomethylation and transcriptional upregulation (hypo-up). (*n* = 3; control, *n* = 4; DAC, reflecting variability in disease induction). Abbreviations: DAC, decitabine; RRBS, reduced representation bisulfite sequencing; RNA-seq, RNA sequencing; DMCs, differentially methylated cytosines; FC, fold change; log₂FC, log₂ fold change; z-score, standardized expression value

DAC improved SpA and suppressed lymphocyte activity in the spleen of SpA mice. To investigate the epigenetic mechanisms underlying this effect, RRBS and RNA-seq were performed on Achilles tendon tissue, including the tendon insertion site—site of inflammation. Integrated RRBS and RNA-seq analysis revealed that DAC administration predominantly induced genes exhibiting both hypomethylation and transcriptional upregulation (hypo-up) (Fig. 5a). As DAC typically induces genomic hypomethylation and upregulates target gene expression, we examined genes exhibiting both hypomethylation and transcriptional upregulation (hypo-up) that are known to regulate Th17 activation. Within DMC analysis, we found that *Vipr1*, a gene associated with Th17-related pathways, was identified as a representative target showing concordant hypomethylation and increased expression (Fig. 5b) [23, 24]. In contrast, differentially Methylated Regions (DMR) analysis yielded only a few significant methylation and transcriptomic changes, which were not directly associated with T-cell functions functions (Supplementary Fig. 3). Thus, among the genes exhibiting coordinated hypomethylation and transcriptional upregulation, *Vipr1* was selected for further validation because it acts as a negative regulator of Th17-mediated inflammation by shifting the T-cell balance away from a Th17 phenotype, thereby exerting a protective role in autoimmune pathogenesis [25].

### DAC upregulates VIPR1 and suppresses IL-17 A in the Achilles enthesis

**Fig. 6:**
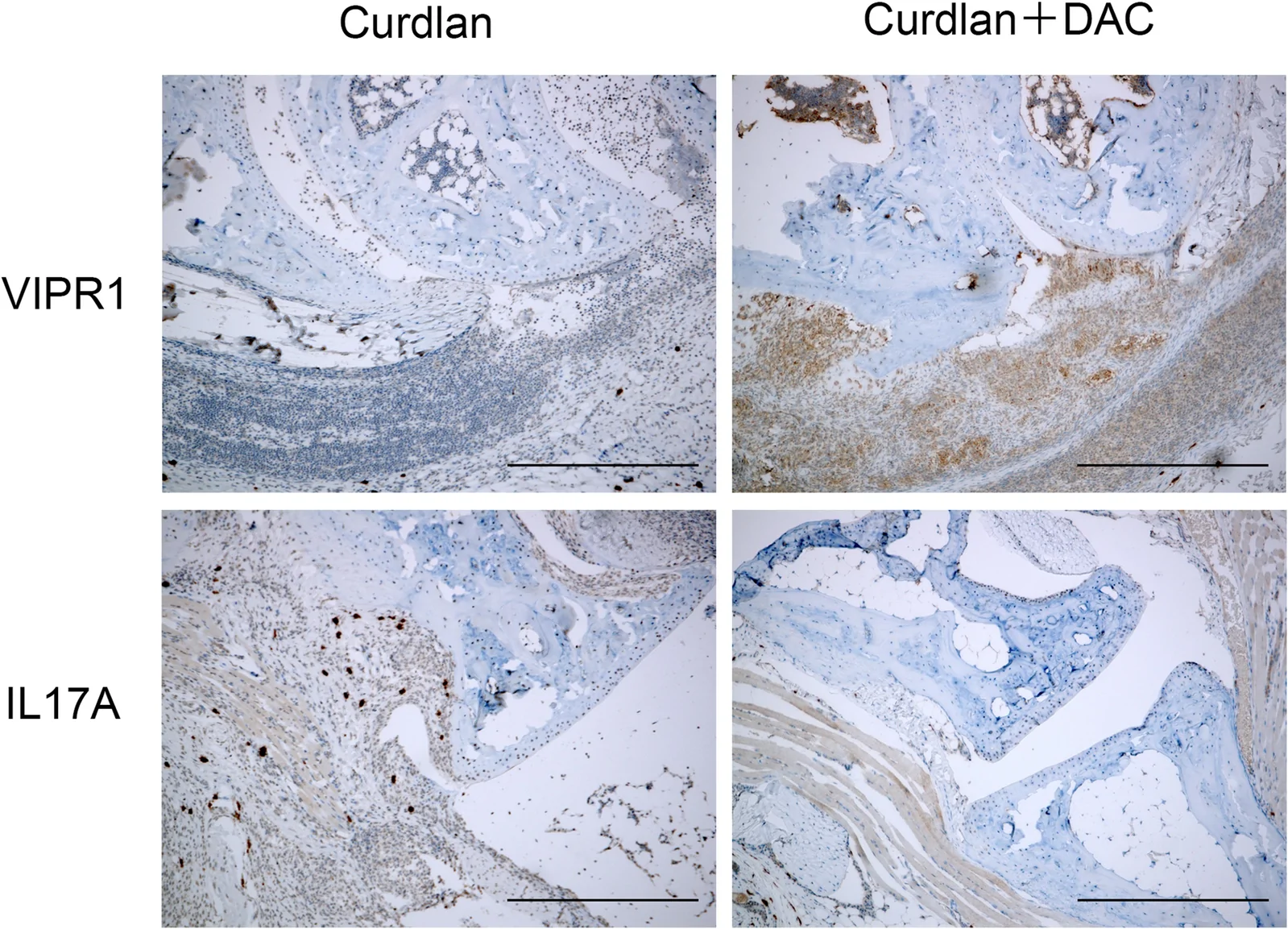
DAC upregulates VIPR1 and suppresses IL-17A in the Achilles enthesis. Representative images of ankle enthesis sections stained for VIPR1 (upper panels) and IL-17 A (lower panels) in vehicle control and DAC-treated mice. Immunoreactivity was visualized using DAB with hematoxylin counterstaining. Scale bar = 500 μm. No quantitative analysis was performed

Protein expression of VIPR1 and IL-17A at the enthesis was assessed by immunohistochemical staining. Representative images are shown in Fig. 6. In the vehicle-control group, IL-17A expression was elevated in the Achilles tendon, and despite pronounced lymphocyte infiltration, VIPR1 expression was reduced throughout the tissue. In contrast, DAC-treated mice exhibited suppressed IL-17A expression and increased VIPR1 expression.

## Discussion

In this study, we demonstrated for the first time that the DNA methylation inhibitor DAC significantly ameliorates SpA-like disease in the SKG mouse model. DAC administration reduced arthritis severity in the limbs and tail, and histopathological analyses revealed marked improvement in synovial hyperplasia, inflammatory cell infiltration, bone erosion, cartilage loss, and peripheral enthesitis. These therapeutic effects were accompanied by suppression of IL-17 production during the early phase of disease and modulation of regulatory T-cell–associated immune regulation, indicating that DAC exerts both systemic and local anti-inflammatory effects in this murine model of SpA.

Our results are consistent with those of previous studies demonstrating that DNA methylation inhibitors exert therapeutic effects in experimental models of autoimmune diseases, including collagen-induced arthritis [12, 13]. Mechanistically, DAC suppresses Th1 and Th17 differentiation while promoting FOXP3 expression, thereby inducing and restoring regulatory T-cell function [14, 16]. These observations provide a mechanistic framework supporting the immunomodulatory effects observed in the present SpA model.

IL-17–driven inflammation is a central pathogenic mechanism in SpA. In this study, DAC significantly suppressed IL-17 production by T cells during the early phase of disease (weeks 4–8 after induction). This effect was observed both in vivo and in vitro in splenic and activated CD4⁺ T cells, respectively, suggesting that DAC can directly modulate Th17 responses independently of the inflammatory microenvironment. Given the established role of IL-17 in SpA pathogenesis and the clinical efficacy of IL-17–targeted therapies, suppression of pathogenic Th17 responses likely represents a key mechanism underlying the therapeutic effects of DAC. Interestingly, DAC did not uniformly suppress all inflammatory cytokines. In particular, IFN-γ production was increased at the early phase (week 4), while IL-17 was suppressed. These findings suggest that DAC may exert selective immunomodulatory effects rather than broad suppression of inflammatory responses. The increase in IFN-γ may reflect altered T-cell activation or differentiation states during early immune modulation, although its precise role remains unclear.

In contrast, DAC did not markedly alter the overall frequency of Tregs. However, PD-1 expression on Tregs was significantly reduced at week 12 in DAC-treated mice. Previous studies in rheumatoid arthritis have demonstrated that chronic inflammation and aging induce PD-1 overexpression on Tregs, leading to impaired suppressive function [26]. In SpA, although reports regarding Treg numbers are inconsistent, accumulating evidence suggests that qualitative defects in Treg function contribute to disease pathogenesis [27–29]. Therefore, the reduction in PD-1 expression observed in this study may be associated with altered regulatory T-cell function rather than a direct mechanistic effect.

To explore the epigenetic mechanisms underlying these immunological effects, we performed integrated methylome–transcriptome analyses using Achilles tendon tissue containing the enthesis, a key site of inflammation in SpA. This analysis revealed that DAC induced both hypomethylation and transcriptional upregulation of *Vipr1*, which encodes the receptor for vasoactive intestinal peptide (VIP). *Vipr1* was selected as a representative gene based on integrative methylome–transcriptome analysis focusing on genes associated with T-cell–related pathways. VIP–VIPR1 signaling suppresses Th17 differentiation and IL-17 production while enhancing regulatory T-cell function [23, 24]. Notably, serum VIP levels are reduced in patients with severe ankylosing spondylitis, suggesting that impaired VIP–VIPR1 signaling may be associated with disease activity [30]. These findings suggest that DAC may exert part of its anti-inflammatory effect through epigenetic modulation of *Vipr1* in this model, thereby restoring an endogenous anti-inflammatory pathway.

The enthesis is a primary anatomical site of inflammation in SpA, where mechanical stress, innate immune activation, and IL-17–mediated immune responses converge [31]. Immunohistochemical analyses demonstrated increased VIPR1 and decreased IL-17 expression at the enthesis in DAC-treated mice, suggesting that DAC may influence not only systemic immune responses but also the local inflammatory microenvironment in this model. Enhanced VIP–VIPR1 signaling at the enthesis may contribute to suppression of local IL-17 production, thereby attenuating enthesitis. In addition, recent studies have shown that VIP contributes to tendon repair by regulating local immune homeostasis, including macrophage polarization toward an anti-inflammatory phenotype and enhancement of tissue regeneration [32]. Although a direct causal relationship between VIPR1 upregulation and IL-17 suppression was not demonstrated, the observed spatial association supports a potential mechanistic link between epigenetic regulation of the VIPR1 pathway and IL-17–driven inflammation in SpA.

From a clinical perspective, these findings provide insight into epigenetic mechanisms underlying SpA and suggest that targeting DNA methylation may represent a potential therapeutic strategy, although validation in human disease is required. Although TNF and IL-17 inhibitors have substantially improved SpA management, a subset of patients exhibits inadequate responses or experiences adverse effects during long-term treatment. Epigenetic agents such as DAC, which target upstream immune-regulatory mechanisms rather than individual cytokines, may therefore offer broader immunomodulatory potential. Given that aberrant DNA methylation patterns have been implicated across multiple autoimmune diseases, the therapeutic relevance of DAC may extend beyond SpA [10, 11]. DAC is clinically used as a hypomethylating agent for myeloid malignancies, including acute myeloid leukemia and myelodysplastic syndromes. However, its use in inflammatory or autoimmune diseases has not been established in humans. Notably, low-dose DAC has been evaluated in patients with immune thrombocytopenia, where restoration of T-cell homeostasis and clinical responses have been reported in some cases [33]. In addition, DNA methylation inhibitors have shown therapeutic potential in experimental models of autoimmune arthritis [34].

These findings suggest that epigenetic modulation may represent a potential therapeutic strategy, although further studies are required to determine its safety and applicability in inflammatory arthritis.

This study had some limitations. First, the sample size was relatively small, and larger studies are required for validation. Second, this study was conducted using a murine model of SpA, and therefore the translatability of these findings to human disease remains uncertain. Third, DAC administration was initiated at disease induction; thus, its efficacy in established disease remains unclear. Forth, potential cytotoxicity and the risk of resistance were not fully evaluated. Fifth, the immunohistochemical evaluation of VIPR1 and IL-17 expression was qualitative; quantitative analyses and cell-type–specific localization will be necessary to further elucidate the cellular targets of DAC within the enthesis. Finally, although *Vipr1* was identified as a candidate gene showing coordinated hypomethylation and transcriptional upregulation, a direct causal relationship between VIPR1 regulation and disease amelioration was not demonstrated in this study. Further mechanistic studies will be required to clarify its functional role in SpA.

## Conclusion

DAC suppressed Th17 responses, modulated regulatory T-cell–associated immune regulation, and induced epigenetic and transcriptional changes in inflamed tissues in a murine model of SpA. The observed increase in VIPR1 expression and concomitant reduction in IL-17 expression at the enthesis support the concept that DAC exerts both systemic and local anti-inflammatory effects. Despite unresolved concerns regarding toxicity and long-term safety, these findings support further investigation of DNA methylation inhibitors as potential epigenetic therapies for SpA and other autoimmune diseases.

## Supplementary Information

Supplementary Material 1: Supplementary Figure 1. Flow cytometry gating strategy. Representative gating strategies used for flow cytometric analysis: (a) Intracellular cytokine staining: Lymphocytes were gated based on forward and side scatter (FSC-A/SSC-A), followed by CD45⁺ and CD3ε⁺ T cells. CD4⁺ and CD8⁺ subsets were identified, and IL-17A⁺ cells were defined as Th17 cells. (b) Treg staining: Lymphocytes were gated on CD45⁺ and CD3ε⁺ populations, followed by identification of CD4⁺CD25⁺Foxp3⁺ regulatory T cells. PD-1 expression was analyzed within the Treg gate. All gates were determined using fluorescence minus one FMO and isotype controls. Representative plots are shown. Abbreviations: FSC-A, forward scatter area; SSC-A, side scatter area; CD, cluster of differentiation; IL-17A, interleukin-17A; Th17, T helper 17; Treg, regulatory T cell; PD-1, programmed cell death protein 1; FMO, fluorescence minus one.

Supplementary Material 2: Supplementary Figure 2. Differential effects of DAC on IFN-γ and TNF-α production. The frequency of cytokine-producing cells was quantified within each subset. (a) Intracellular cytokine staining showing the frequency of IFN-γ positive cells among CD3⁺, CD4⁺, and CD8⁺ T cells. (b) Intracellular cytokine staining showing the frequency of TNF-α positive cells among CD3⁺, CD4⁺, and CD8⁺ T cells. (*n* = 8; weeks 4 and 8, *n* = 10; week 12) Data represent mean ± SD. Groups were compared using Welch’s t-test as appropriate. *p < 0.05. Abbreviations: DAC, decitabine; TNF-α, tumor necrosis factor alpha; IFN-γ, interferon gamma.

Supplementary Material 3: Supplementary Figure 3. Methylated Regions (DMR) analysis yielded only a few significant methylation and transcriptomic changes. Genome-wide DMR analysis identified regions exhibiting significant DNA methylation changes (mean Δmethylation ≥25%, *p* < 0.05). Integration with transcriptomic data (|log₂FC| > 1, *p* < 0.05) identified genes showing coordinated methylation and expression changes. (*n* = 3; control, *n* = 4; DAC, reflecting variability in disease induction). Abbreviations: DMR, differentially methylated region; DAC, decitabine.

## Data Availability

The datasets generated in this study have been deposited in the NCBI Sequence Read Archive (SRA) under BioProject accession number PRJNA1442296 and will be publicly available upon publication.

## Abbreviations

SpA: Spondyloarthritis
DAC: Decitabine
IL: Interleukin (including IL-17 and IL-23)
TNF: Tumor necrosis factor
Treg/Tregs: regulatory T cell(s)
Th17: T helper 17
SKG: SKG mouse strain
PBS: Phosphate-buffered saline
HE: Hematoxylin–eosin
TB: Toluidine blue
RRBS: Reduced representation bisulfite sequencing
RNA-seq: RNA sequencing
DMC(s): Differentially methylated cytosine(s)
VIPR1: Vasoactive intestinal peptide receptor 1
